# ‘The Plastic Nile’: First Evidence of Microplastic Contamination in Fish from the Nile River (Cairo, Egypt)

**DOI:** 10.3390/toxics8020022

**Published:** 2020-03-25

**Authors:** Farhan R. Khan, Yvonne Shashoua, Alex Crawford, Anna Drury, Kevin Sheppard, Kenneth Stewart, Toby Sculthorp

**Affiliations:** 1 Department of Science and Environment, Roskilde University, Universitetsvej 1, P.O. Box 260, DK-4000 Roskilde, Denmark; 2 Environmental Archaeology and Materials Science, National Museum of Denmark, IC Modewegsvej Brede, DK-2800 Kongens Lyngby, Denmark; Yvonne.Shashoua@natmus.dk; 3 Sky News International, Grant Way, Islwwroth, Middlesex TW7 5QD, UK; Alex.Crawford@sky.uk (A.C.); annamc25@hotmail.com (A.D.); Kevin.Sheppard@sky.uk (K.S.); Kenneth.Stewart@sky.uk (K.S.); Toby.Sculthorp@sky.uk (T.S.)

**Keywords:** microplastics, freshwater, Africa, ingestion, Nile tilapia (*Oreochromis niloticus*), catfish (*Bagrus Bajad*), fibers, ATR-FTIR spectroscopy

## Abstract

The presence of microplastics (MPs) in the world’s longest river, the Nile River, has yet to be reported. This small-scale study aimed to provide the first information about MPs in the Nile River by sampling the digestive tracts of two fish species, the Nile tilapia (*Oreochromis niloticus*, *n* = 29) and catfish (*Bagrus bayad*, *n* = 14). Fish were purchased from local sellers in Cairo, and then their gastrointestinal tracts were dissected and examined for MPs. Over 75% of the fish sampled contained MPs in their digestive tract (MP prevalence of 75.9% and 78.6% for Nile tilapia and catfish, respectively). The most abundant MP type was fibers (65%), the next most abundant type was films (26.5%), and the remaining MPs were fragments. Polyethylene (PE), polyethylene terephthalate (PET) and polypropylene (PP) were all non-destructively identified by attenuated total reflectance Fourier transform infrared spectroscopy. A comparison with similar studies from marine and freshwater environments shows that this high level of MP ingestion is rarely found and that fish sampled from the Nile River in Cairo are potentially among the most in danger of consuming MPs worldwide. Further research needs to be conducted, but, in order to mitigate microplastic pollution in the Nile River, we must act now.

## 1. Introduction

Microplastic pollution (MPs, defined as <5 mm in size) has been found in all compartments of the aquatic environment—water, sediment and the animals that inhabit them. Microplastics have been found in the depths of the ocean [1,2] and in remote mountain locations [3,4]. However, some significant knowledge gaps relating to the presence and abundance of MPs across our planet remain, both in terms of aquatic habitat and geographical location. Most of the scientific research into MP pollution still focuses on the marine environment, and less is known about MPs in freshwaters [5]. MP research is largely concentrated in Europe and North America, and is under-represented in Africa, Asia and South America [5]. Despite the fact that the primary focus of MP research has been in the marine environment, there has been increasing interest in determining the presence MPs in river systems. Countless people depend on rivers for a variety of vital functions, and, furthermore, rivers have been shown as major pathways for the transport of substantial amounts of plastic debris and MPs from land-based sources to the oceans [6,7]. Thus, in recent years, MPs have been documented in the water, sediment and biota of the some of the world’s major river systems; the Thames (UK) [8,9], the Seine (France) [10], the Rhine (Germany) [11] and the Danube (Austria) [12] in Europe; the Amazon in South America [13,14]; the Yangzte in Asia (China) [15,16]; and the St. Lawrence in North America [17]. One notable river not present on this list, arguably the most famous river of all, is the Nile River. The aim of this small-scale study was to rectify this knowledge gap and provide the first information about MPs in the Nile River.

**Figure 1:**
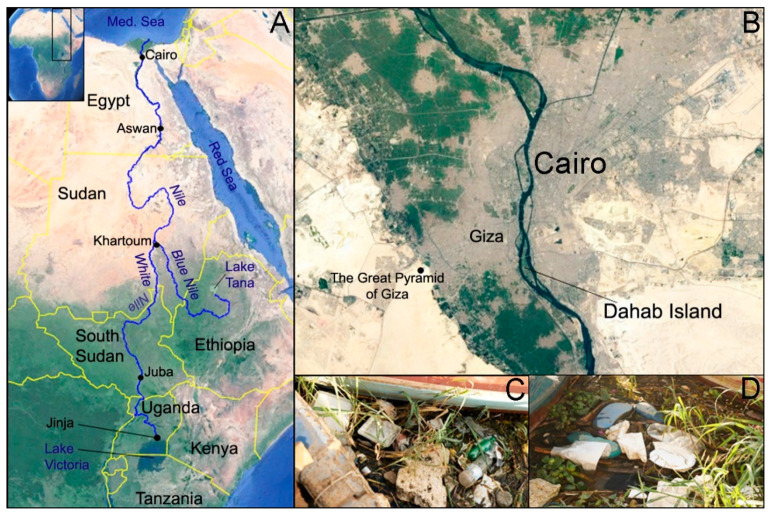
Path of the Nile River starting at Lake Tana (Blue Nile) and Lake Victoria (White Nile), respectively, before meeting at Khartoum and flowing toward the Mediterranean Sea via Cairo, the area of the present study (**A**). Fish were caught around Dahab Island in the Nile River at Cairo and purchased from markets located along the Nile Corniche on the east bank of the Nile opposite Dahab Island (**B**). Examples of plastic litter found close to Dahab Island and along the Nile (**C**,**D**). (Map source: Google Earth (2019) with the path of the Nile River overlaid from shapefiles [30]).

The Nile River is the longest river in the world at 6693 km [18], and it is the only permanent river to cross the Sahara Desert [19]. The two major tributaries of the Nile are the White Nile and the Blue Nile (Figure 1). While the latter’s origin in Lake Tana in Ethiopia is well known, the origin of the longer White Nile is debatable, but it is considered to be a tributary of the Kagera River which flows into Lake Victoria [19]. The White Nile tributary then leaves the African Great Lake at the Ugandan city of Jinja, flows northwards through South Sudan and its capital Juba, and then flows on through Sudan where the White and Blue Nile tributaries join at the Sudanese capital Khartoum, which has an estimated population of close to 6 million people [20]. The Nile continues north into Egypt, passing through Aswan before flowing through Cairo, which has a population estimated to be approximately 20 million people by 2020 [20]. The Nile River drains into Mediterranean Sea via the Nile Delta (Figure 1). Whilst the Nile flows through numerous countries, it has always been inextricably linked to Egypt and has been described as the “donor of life to Egypt” [21]. The role of the Nile in establishing the Ancient Egyptian civilizations cannot be understated, and the historic dependence on the Nile continues today through agriculture, transport, fishing and tourism [19]. However, the adverse impacts of plastic and MPs could be a pervasive threat that have not yet been researched. Here, we investigate the presence of MPs by looking within the gastrointestinal (digestive) tracts of two fish species found in the Nile River, the Nile tilapia (*Oreochromis niloticus*) and catfish (*Bagrus Bajad*).

Sampling the digestive tracts of resident fish populations has become a recognized approach by which to assess the extent of MP pollution in the environment. Examples of this type of study have found MPs in the gastrointestinal tracts of fish from the English Channel [22], French freshwater systems [23], the Mediterranean coast of Turkey [24], the Amazon River estuary [13] and Lake Victoria [25]. This last study was first to document the presence of MPs in African freshwaters and was conducted in the largest of Africa’s Great Lakes. Nile perch (*Lates niloticus*) and Nile tilapia were selected for their economic and ecological importance and were purchased from the local market in the Mwanza region of Tanzania. Gastrointestinal tracts were dissected and then digested in a strong alkaline solution to isolate MPs. In total, suspected plastics were recovered from the gastrointestinal tracts of 11 perch (55%) and seven tilapia (35%), and they were confirmed in 20% of each fish species (i.e., four individuals) by attenuated total reflectance Fourier transform infrared (ATR-FTIR) spectroscopy, the definitive analytical technique for identifying the chemical composition of plastic polymers. A variety of polymers were recovered from the fish including polyethylene, polyurethane and polyester, which are found in packaging, clothing, and food and drink containers. The likely sources of these plastics are considered to be human activities linked to fishing and tourism, as well as urban waste [25]. Though limited in the number of fish used, the Lake Victoria study exemplified how a small-scale investigation could be conducted and provide an early indications of MP pollution [26].

The present study was conducted with fish caught in the center of Cairo and used the same methods as in Lake Victoria. Nile tilapia were once again utilized, and the second species used was the catfish, *Bagrus Bayad*. Nile tilapia typically inhabit shallow waters and are omnivorous with a diet consisting of plankton and smaller fish [27], whereas the much larger catfish are exclusively piscivorous, living and feeding near the bottom of the water column [28]. The presence of MPs in these two species of differing niches and feeding habitats could provide additional information by which to better understand the fate of MPs in freshwater rivers.

This study was conceived as part of the documentary ‘The Plastic Nile’ produced by Sky News International. It was conducted within a relatively short period of time (five days) and under a degree of confidentiality that is not usual for scientific investigations. The reason for this is that criticism of the Nile is often negatively received by the authorities, as evidenced by the case of Egyptian singer Sherine Abdel Wahab, who was sentenced to six months imprisonment for commenting on the cleanliness of the Nile River [29]. Thus, the work was performed in a laboratory within the greater Cairo area, but no further details are provided as to not identify our hosts. Moreover, they have requested not to be added as co-authors to this work, but we greatly acknowledge and appreciate their collaboration, without which this work would not have been possible.

## 2. Materials and Methods

### 2.1. Study Area and Fish

In December 2018, twenty-nine Nile tilapia (*Oreochromis niloticus*) and fourteen catfish (*Bagrus bayad*) were purchased from local sellers situated along the Nile Corniche in Cairo, where fish are caught and sold daily. The fish were caught in the vicinity of Dahab Island, which is located in the heart of Cairo and close to The Great Pyramid of Giza (Figure 1). Fish were purchased whole without the prior removal of their gastrointestinal tracts (i.e., they were not gutted) and transported to the laboratory, where they were promptly refrigerated at 4 °C. The gastrointestinal tracts of each fish were removed within 24 h of arrival in the laboratory. Prior to dissection, all fish were measured and weighed. Nile tilapia (*n* = 29) had an average weight and length of 129.7 ± 59.0 g and 17.7 ± 2.5 cm, respectively, and the catfish (*n* = 14) were considerably larger at 1496.4 ± 849.2 g and 56.5 ± 18.9 cm. The catfish weight exceeded the limit of the digital laboratory balance and were therefore weighed on an analog kitchen scale. The heaviest catfish was just over 3000 g. The length and weight of each individual fish used in this study can be found in Table S1 (Supplementary Information).

### 2.2. Tissue Digestion and MP Extraction

The methods for dissection and digestion, as well as the subsequent isolation of MPs, were conducted as previously described by Biginagwa et al. (2015) [25]. For each fish, the entire gastrointestinal tract from buccal cavity to anus was dissected following a longitudinal incision of the abdomen. All efforts were made to eliminate sample contamination with the thorough cleaning of dissection utensils with 70% ethanol and lint-free paper between dissections. A preliminary examination was made of each gastrointestinal tract, and, in the case of catfish, undigested smaller fish were removed. Dissected tissues were placed in 250 or 500 mL conical flasks, to which 10 M NaOH was added in a 5:1 (*w*/*v*) ratio. The NaOH digestion (60 °C for 24 h) was used to isolate plastic litter from the organic tissue. This method, which involves a strong basic solution, has been shown to digest organic matter with an efficacy of >90% [15,31] whilst importantly having negligible impact on the plastics, especially when compared to strong acid digestion, which can discolor or degrade plastics. Post-digestion, the plastics and a minimal amount of partially digested tissue were rinsed from the NaOH through 250 µm mesh stainless steel sieves under running water and then placed on filter paper (Whatman^®^ Grade 540, Hardened Ashless Filter Paper, 90 mm diameter, GE Healthcare Life Sciences, UK) to dry under a fume hood. The dried samples were then tightly wrapped within the filter papers, sealed in 50 mL falcon tunes, and transported to the laboratory at Roskilde University (Denmark).

In the laboratory, each sample was examined under a light dissection microscope (×40). MPs were initially visually identified due to their possession of unnatural coloration (such as bright blue) and/or unnatural shapes (such as fragments with sharp edges) [32,33]. A secondary visual inspection was made in which each suspected item was required to possess the following criteria as described by Nor and Obbard (2014) [34] and Horton et al. (2018) [35]: (1) no visible cellular or organic structures, (2) unsegmented, (3) fibers of homogenous width, (4) the appearance of homogenous material, (5) fibers that remained intact if pulled with tweezers, and (6) flexible, but not brittle. These two steps were used to document the presence of MPs. MPs found per fish were enumerated and categorized as fibers, fragments, films, pellets, foams or beads, according to Tanaka and Takada (2016) [36]. Pictures of the suspected MPs were taken with an Olympus uc90 digital camera mounted on an Olympus SZ61 microscope. A subsample of MPs was analyzed by Fourier transform infrared spectroscopy to verify the visual identification based on its chemical structure.

### 2.3. Fourier Transform Infrared Spectroscopy

The chemical composition of a representative sample of MPs was non-destructively identified by attenuated total reflectance Fourier transform infrared (ATR-FTIR) spectroscopy. ATR-FTIR has become a standard analytical technique for identifying the chemical composition of samples larger than 0.5 mm. However, owing to this size constraint, the representative sample analyzed was made from those plastic particles that were at least 0.5 mm in one dimension. In total, 10% of the collected MPs were identified by ATR-FTIR. Scans were run at a resolution of 2 cm^−1^ between 4000 and 650 cm^−1^ on a Bruker Alpha-p FTIR spectrometer (Bruker, Billirica, MA, USA) fitted with a diamond single-bounce internal reflectance element. Spectra were compared with reference standards run on the same instrument and processed using Opus software supplied by Bruker. To make a positive polymer identification, we used the approach advocated by Frias et al. (2016) [37] and the European Union expert group on marine litter (Subgroup on Marine litter (TSG-ML)) to only accept matches of >70% similarity to the reference library samples [38].

### 2.4. Quality Assurance Measures

Neoprene gloves and cotton lab coats were worn during the dissection, filtering, and microscope processes in order to avoid contamination. All glassware used was washed and rinsed with distilled water. During the process to isolated MPs, six background samples (petri dishes containing filter papers) were placed in the lab space. This included around the dissection area and under the fume hood whilst the samples dried to account for any airborne MPs. The references were transported along with the samples back to Denmark. All background samples were systematically examined under light dissection microscope all with the other samples. Only one black fiber was found in the reference petri dishes.

### 2.5. Analysis

The number of individuals containing MPs from each species was expressed as the frequency of occurrence of MPs (FO%), as follows: FO% = (Ni/N) × 100, where Ni is the number of digestive tracts that contained MPs and N is the total number of gastrointestinal tracts examined [13]. Differences in FO% and the average number of items found per individual between Nile tilapia and catfish were assessed by an unpaired *t*-test (SPSS version 22 (SPSS statistics for windows, SPSS Inc., Chicago, IL, USA)).

## 3. Results and Discussion

### 3.1. Abundance of MPs in Fish Gastrointestinal Tracts

Over 75% of the 43 fish sampled in this study contained MPs in their gastrointestinal tract (33 out of 43, FO = 76.7%). From these 33 fish, a total of 211 items of plastic were recovered. The highest number of MPs recovered from a single fish was 20 individual items, which were found in a Nile tilapia (sample #15, Table S1). The two species showed similar levels of MP prevalence, with FO values of 75.9% and 78.6% for Nile tilapia and catfish, respectively. Of the 22 out of 29 Nile tilapia that contained MPs in their gastrointestinal tract, 164 MPs were recovered. Thus, each tilapia that contained MPs contained, on average, 7.5 ± 4.9 items. Forty-seven MPs were recovered from the 11 out of 14 catfish with MPs in their digestive tracts, with each individual containing MPs having an average burden of 4.7 ± 1.7 items (Table 1). This difference between 7.5 ± 4.9 and 4.7 ± 1.7 items per fish was significant (*p* = 0.046; unpaired *t*-test), thus suggesting that Nile tilapia either consume more MPs than catfish or better retain them within their digestive system. As Nile tilapia inhabit shallower waters closer to the surface, they may be more likely to come into contact with MPs than the bottom-dwelling catfish. Moreover, the omnivorous diet of the tilapia which contains plankton may mean that it is more likely to mistake plastic items for food rather than the strictly piscivorous catfish. Any species differences should be treated with caution due to the limited numbers of fish involved in this study. However, a comparison of feeding type did find that omnivorous fish with their diverse diets and higher feeding rates were more likely to contain MP fibers than either herbivorous or carnivorous species [39].

The frequency of occurrence in fish sampled from the Nile River at Cairo appears to generally higher than those reported from other locations. Fish sampled from the marine environment have wide ranging values; at the low end of the scale, only 2.6% of fish sampled from the North Sea [40] and only 5.5% of fish in the North and Baltic Seas [41] contained MPs. Elsewhere, the prevalence of MPs in different fish species has been determined to be 19.8% of fish from the Portuguese coast [42], 37% from the English Channel [22], and 68% from the Balearic Islands [43]. The Mediterranean Sea, into which the Nile River flows, is perhaps the most valid marine comparison to be made. An investigation of MP prevalence in fish from the Turkish waters of the Mediterranean Sea found that 41% of sampled fish representing 28 species contained MPs [24]. In Spanish and Mediterranean coastal waters, 17.5% of examined fish contained MPs in their digestive tracts, with the highest occurrence in red mullet (18.8%) [44]. The larger pelagic fish of the Mediterranean—swordfish, Bluefin tuna and albacore—had occurrences of 12.5%, 32.4% and 12.9%, respectively [45].

In comparing studies, it is important to make relevant comparisons. For the Nile River, the two most relevant comparisons are studies in which fish were sampled from freshwater rivers or conducted in locations with similar sized urban populations. For each of these parameters, there are only a handful of relevant findings to compare against. Perhaps the only comparable river to the Nile in the world is the Amazon, and the two have both been described as the world’s longest river. However, the MP prevalence in fish from the Amazon estuary and northern coast of Brazil is markedly less than those from the Nile, as MPs have only been found in 26 out of 189 examined gastrointestinal tracts (13.8%) across 14 different species [13]. Fish sampled from French and Belgian freshwater river systems have been shown to have an overall FO of only 12% and 9%, respectively [23,46] and, of the fish sampled from the River Thames (UK), 33% contained MPs [35]. However, whist the Thames is considered to be a major river and the city of London has a sizeable population, the sampling sites for this study were situated outside of the largest urban areas. Research conducted in Tokyo Bay (Japan) with a population in the bay’s drainage area of 29 million people found that 77% of Japanese anchovies contained MPs in their digestive tracts [36]. Very few studies have reported higher occurrences, but in the Río de la Plata [47] and Bahía Blanca [48] estuaries in Argentina, MPs have been found in 100% of the sampled fish. Notably, the study by Pazos et al. (2017) [47] reported an average number of MPs per fish as 18.5 ± 18.9, whilst more typical numbers were found to be between 2 and 10. Our results predominantly fit in this range.

Comparing a wide range of aquatic habitats and fish species, it appears that the frequencies of occurrence for MPs in Nile tilapia and catfish sampled from the urban waters of Cairo are amongst the higher levels reported in the scientific literature.

### 3.2. Characteristics and Identification of Polymer Types

**Figure 2:**
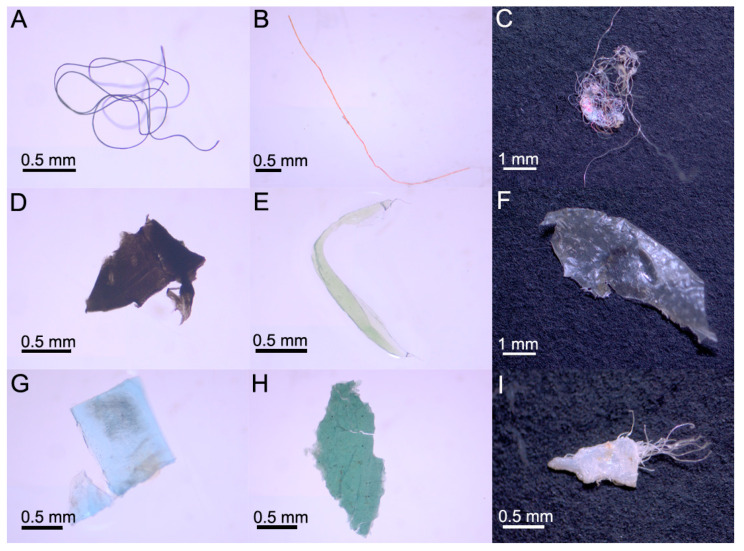
Examples of MPs found within the gastrointestinal tracts of Nile Tilapia and Catfish: fibers (blue polyethylene (PE) (**A**), Red polyethylene terephthalate (PET) (**B**), and PET fiber knot (**C**)), films (black (**D**), green (**E**) and transparent (**F**) films were all identified as PE), and fragments (light blue polypropylene (PP) (**G**), blue-green PP (H) and white PET (**I**)).

Fibers were the most commonly found MP type, accounting for 65.3% and 61.7% of items found in the gastrointestinal tracts of Nile tilapia and catfish, respectively. Films were the second most abundant, with 25.6% (Nile tilapia) and 29.8% (catfish), and fragments made up 8.5% of the recovered items in both species (Table 1). Pellets, foams and beads were not found. Black and red colored MPs were most abundant for fibers and films (black > red > blue > green > other > transparent for fibers and black > red > transparent > green > blue for films). Fragments were predominantly blue (blue > black > transparent). The full dataset for MP number, type and color per individual can be found in Table S1 (Supplementary Information). A selection of the MPs recovered from the digestive tracts of the sampled fish can be found in Figure 2.

The dominance of fibers as the most abundant MP type is well founded in the literature. Fibers constituted the greatest proportion of MPs in fish from the Río de la Plata estuary (96%) [47] and in the North Pacific Gyre (94%) [49]. The studies by Neves et al. (2015) [42], Bellas et al. (2016) [44] and Arias et al. (2019) [48] also reported greater numbers of fibers compared to other MP types within piscine digestive tracts. The reason for fibers being so common has been attributed to their diverse origin. Fibers may result from the degradation of clothing items, furniture and fishing gear. Washing a single item of synthetic clothing may release approximately 2000 fibers [50].

**Figure 3:**
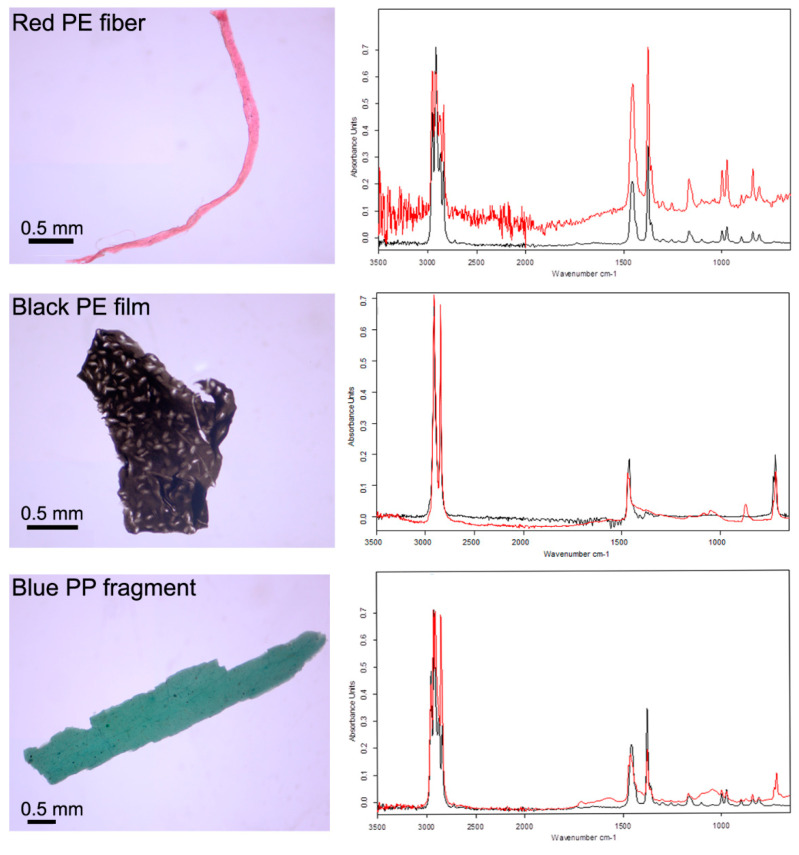
Fourier-transform infrared (FTIR) spectra of representative samples identified as polyethylene (PE) (both red fiber and black film) and polypropylene (PP) (blue-green fragment). Sample spectra are shown as red and compared to known reference samples in black. Samples exhibited weathering and oxidation, as shown by the presence of additional broad peaks, e.g., at wavelengths of 1620–1750 cm^−1^ and 1100 cm^−1^ in the spectra of the blue PP fragment.

ATR-FTIR spectroscopy was used to verify the visual selection of MPs. All of the analyzed MPs (approximately 10% of the 211 recovered MPs) were plastic polymers. Polyethylene (PE), polyethylene terephthalate (PET), and polypropylene (PP) were all found (Figure 2 and Figure 3) in keeping with other studies. These polymers are used in a multitude of consumer products from clothing, to packaging, to plastic bags, as well as in fishing nets and ropes. Given the diverse usage of commonly found polymers, it is not possible to directly attribute the presence of MPs within fish digestive tracts to any specific source.

ATR-FTIR also revealed that a number of the MPs had undergone a degree of weathering and chemical oxidation (Figure 3). For example, the spectrum of the blue PP fragment had developed additional broad peaks at 1620–1750 cm^−1^ and 1100 cm^−1^ that may be attributed to degradation compared to the pristine PP reference material. MPs in African freshwaters, as in other equatorial locations, are likely to degrade faster than in more temperate conditions because reactions such as photolysis, thermo-oxidation and photo-oxidation are accelerated by intensive UV light [51]. The implications of weathering are not well understood, but environmentally-aged or degraded particles may have the rate at which they sink though the water column altered, which in turn may effect which organisms they meet. Furthermore, weathered MPs maybe more susceptible to biofouling (the attachment of microorganisms to the surface of the MP) or the adsorption of other environmental pollutants such as hydrophobic, organic contaminants or trace metals [52,53].

### 3.3. Future Considerations

Our study provides the first assessment of MP pollution in the Nile River. Over 75% of the sampled fish contained at least one item of plastics in their gastrointestinal tract, and five of the Nile tilapia contained 10 MPs or more (Table S1, Supplemental Information). Comparisons across studies from marine and freshwater environments show that this level of MP ingestion is rarely found and that fish sampled from the Nile River in Cairo are potentially among the most in danger of consuming MPs on the planet. However, ours was only a small-scale study, and, although this preliminary data present a worrying scenario, we likely underestimate the true extent of plastic ingestion by Nile tilapia and catfish. Here, we were constrained by time, fish availability and the size-selected MPs of above 250 µm. Thus, the MPs below this size were not captured, and, based on previous research, this is likely to be a sizeable fraction of environmentally-present MPs [54]. Moreover, smaller-sized plastic items, such as those found in the nano-size range (less than 1 µm), have the potential to cross intestinal barriers and enter fish tissue, which could, in turn, allow MPs to enter the human food chain [55]. Since most fish, including those used in this study, are eaten following the removal of the digestive tract, the presence of larger MPs is less concerning in terms human consumption, but the presence of MPs in fish digestive tracts is in itself a disturbing phenomenon that has been reported from many locations.

As well as being the first to describe the presence of MPs in fish from the Nile, this study is only the second to describe MPs within fish from African freshwaters—the first having been conducted in Lake Victoria [25]. Other research has quantified and characterized the presence of MPs in African freshwaters, such as within gastropods from the Osun River system in Nigeria [56] and within the sediments of the lagoon of Bizerte in Tunisia [57]. However, such studies are scarce, and there is a pressing need for more information regarding the prevalence of MPs in Africa’s inland freshwaters [26].

The present study, as well those few others conducted in Africa, present only a ‘snapshot in time’ of MP pollution in African waters. Based on our preliminary findings from Cairo, but also with the broader perspective encompassing African freshwaters, we suggest that establishing a complete picture of MP pollution along the Nile River should be considered a research priority. This may be through a continuous environmental monitoring program for MP pollution that encompasses water, sediment, and biota, as well as sampling at a number of sites—particularly those with a dense urban population.

The confirmation of MPs in the Nile River is only the first, albeit necessary, step of starting to understand and stop plastic pollution in the world’s longest river. Longer-term research needs to be conducted that also encompasses the impacts of MP pollution on fish populations and its potential transfer to surrounding human populations. Understanding the sources and fate of MPs that enter the Nile is key to mitigating its impacts, but this understanding requires the collaboration of numerous interested stakeholders. The Nile River is part of the human story, and the advent of the ‘plastic age’ may result in impacts on this ancient river that are not yet possible to predict. It may already be too late to prevent such outcomes, but if we are to succeed, we must act without delay.

## Figures and Tables

**Table 1:** Summary of data for Nile tilapia and catfish. The number of individual fish analyzed (n), average weights, and average lengths (± standard deviation) are provided. The frequency of occurrence (in %) describes the number of fish with microplastics (MPs) present in their gastrointestinal tracts. The total numbers of MPs found per species and average per individual are given alongside a breakdown of the different MP types found (in %). The full dataset of MPs found in each individual fish can be found in Supplemental Information (Table S1).

Common Name	Species Name	*n*	Weight (g)	Length (cm)	% FO	Total Number of MPs Found	Mean MPs Per Individual Found with MPs	Type (%)		
								Fibers	Films	Frag.
Nile tilapia	*Oreochromis niloticus*	29	129.7 ± 59.0	17.7 ± 2.5	75.9	164	7.5 ± 4.9	65.3	25.6	8.5
Catfish	*Bagrus Bajad*	14	1496.4 ± 849.2	56.5 ± 18.9	78.6	47	4.7 ± 1.7	61.7	29.8	8.5

## Supplementary Materials

The following are available online at https://www.mdpi.com/2305-6304/8/2/22/s1, Table S1: Full dataset of microplastic number, type and color found in each individual Nile tilapia (A) and catfish (B).
