# Supplementary material for: ‘The Plastic Nile’: First Evidence of Microplastic Contamination in Fish from the Nile River (Cairo, Egypt)
Source: Toxics. 2020 Mar 25;8(2):22. doi: 10.3390/toxics8020022 (PMC7356599; doi:10.3390/toxics8020022)
Supplement: Supplementary file 1 [file toxics-08-00022-s001.pdf]

**Supplementary Materials: ‘The Plastic Nile’: First Evidence of Microplastic Contamination in Fish from the Nile River (Cairo, Egypt)**

**Farhan R. Khan, Yvonne Shashoua, Alex Crawford, Anna Drury, Kevin Sheppard, Kenneth Stewart and Toby Sculthorp**

**Table S1.** Full dataset of microplastic number (#), type and colour found in each individual Nile tilapia (A) and catfish (B).

| A. Nile tilapia (Oreochromis niloticus) | | | | | | | | | | | | | | | | | | | | | | | | |
|---|---|---|---|---|---|---|---|---|---|---|---|---|---|---|---|---|---|---|---|---|---|---|---|---|
| # | Wt | L | Fibers | | | | | | Films | | | | | | Fragments | | | | | | Total | % of total | | |
| | (g) | (cm) | Red | Black | Blue | Green | Trans. | Other | Red | Black | Blue | Green | Trans. | Other | Red | Black | Blue | Green | Trans. | Other | MPs | Fib | Film | Frag |
| 1 | 165.4 | 19.6 | 6 | 4 | | | | 1 | | 1 | | 1 | | | | 4 | | | | | 17 | 65 | 12 | 24 |
| 2 | 135.7 | 18.4 | 6 | 8 | | | | | | | | | | | | | 2 | | | | 16 | 88 | 0 | 13 |
| 3 | 155.2 | 19.7 | 2 | 1 | | 1 | | | | | | | | | | | | | | | 4 | 100 | 0 | 0 |
| 4 | 101 | 17.3 | 1 | 4 | | | | | | 1 | | | | | | | | | | | 6 | 83 | 17 | 0 |
| 5 | 72.08 | 15.1 | | | | | | | | | | | | | | | | | | | 0 | 0 | 0 | 0 |
| 6 | 245 | 22 | | 2 | 2 | | | | | 1 | | 1 | | | | | 4 | | | | 10 | 40 | 20 | 40 |
| 7 | 154.4 | 18.6 | 3 | 1 | 2 | | | | | 1 | | | | | | | | | | | 7 | 86 | 14 | 0 |
| 8 | 118.4 | 18.1 | | | | | | | | 1 | | | | | | | 1 | | | | 2 | 0 | 50 | 50 |
| 9 | 199 | 21.4 | | | | | | | 1 | 5 | 1 | | | | | | | | | | 7 | 0 | 100 | 0 |
| 10 | 103 | 18 | 1 | 3 | 1 | | | | 1 | | | | | | | | | | | | 6 | 83 | 17 | 0 |
| 11 | 137.1 | 19.9 | | 1 | 2 | 1 | | | | | | | 2 | | | | | | | | 6 | 67 | 33 | 0 |
| 12 | 230.3 | 21.7 | | | | | | | | | | | | | | | | | | | 0 | 0 | 0 | 0 |
| 13 | 178.8 | 19.5 | 1 | 4 | | 1 | | | | | | | | | | | | | | | 6 | 100 | 0 | 0 |
| 14 | 83.69 | 15.2 | 12 | 6 | | 1 | | | | | | | | | | 1 | | | | | 20 | 95 | 0 | 5 |
| 15 | 103 | 16.1 | 1 | | 1 | 4 | | | 2 | 4 | | | | | | | | | | | 12 | 50 | 50 | 0 |
| 16 | 303.6 | 22.7 | 1 | | 5 | | | | | 1 | | | | | | | | | | | 7 | 86 | 14 | 0 |
| 17 | 152.6 | 18 | | | | | | | | | | | | | | | | | | | 0 | 0 | 0 | 0 |
| 18 | 93.04 | 16.4 | 1 | | 2 | | | | 2 | | | | | | | | | | | | 5 | 60 | 40 | 0 |
| 19 | 117.9 | 17.4 | | | | | | | | | | | | | | | | | | | 0 | 0 | 0 | 0 |
| 20 | 80.18 | 15.5 | | 1 | | | | | 2 | 3 | | 1 | 1 | | | | | | | | 8 | 13 | 88 | 0 |
| 21 | 87.48 | 15.5 | | 3 | | | | | | | | | 1 | | | | 2 | | | | 6 | 50 | 17 | 33 |
| 22 | 130.7 | 17.7 | 1 | 3 | | | | 1 | 1 | 1 | | | 1 | | | | | | | | 8 | 63 | 38 | 0 |
| 23 | 152.2 | 18.9 | | | | | | | | | | | | | | | | | | | 0 | 0 | 0 | 0 |
| 24 | 87.52 | 15.7 | | | | | | | | | | | | | | | | | | | 0 | 0 | 0 | 0 |
| 25 | 120.5 | 17.4 | | 2 | 1 | | | | | 2 | | | | | | | | | | | 5 | 60 | 40 | 0 |
| 26 | 74.91 | 15.5 | | 1 | | | | | | 2 | | | | | | | | | | | 3 | 33 | 67 | 0 |

| | | | | | | | | | | | | | | | | | | | | | | | |
|---|---|---|---|---|---|---|---|---|---|---|---|---|---|---|---|---|---|---|---|---|---|---|---|
| 27 | 60.97 | 14.5 | | 1 | | | | | | | | | | | | | | | | | 1 | 100 | 0 | 0 |
| 28 | 56.35 | 13.2 | | | | | 1 | | 1 | | | | | | | | | | | | 2 | 50 | 50 | 0 |
| 29 | 62.79 | 14.5 | | | | | | | | | | | | | | | | | | | 0 | 0 | 0 | 0 |

**B. Catfish (Bagrus Bajad)**

| # | Wt (g) | L (cm) | Fibers | | | | | | Films | | | | | | Fragments | | | | | | Total MPs | % of total | | |
|---|---|---|---|---|---|---|---|---|---|---|---|---|---|---|---|---|---|---|---|---|---|---|---|---|
| | | | Red | Black | Blue | Green | Trans. | Other | Red | Black | Blue | Green | Trans. | Other | Red | Black | Blue | Green | Trans. | Other | | Fib | Film | Frag |
| 1 | 880 | 50 | | | | | | | | 2 | | | 1 | | | | | | | | 3 | 0 | 100 | 0 |
| 2 | 960 | 51.5 | | | | | | | | | | | | | | | | | | | 0 | 0 | 0 | 0 |
| 3 | 1380 | 57.9 | | 3 | | | | | | | | | | | | | | | | | 3 | 100 | 0 | 0 |
| 4 | 880 | 42.7 | | 3 | | | | | 1 | 1 | | | | | | | 1 | | | | 6 | 50 | 33 | 17 |
| 5 | 650 | 41.7 | 1 | 3 | 1 | | | | | | | | | | | | | | | | 5 | 100 | 0 | 0 |
| 6 | 490 | 35.8 | | 4 | | | | | | 2 | | | | | | | 2 | | | | 8 | 50 | 25 | 25 |
| 7 | 410 | 35.1 | | | | | | | | | | | | | | | | | | | 0 | 0 | 0 | 0 |
| 8 | 2000 | 66.4 | | | 1 | | | | 1 | 2 | | | | | | | | | | | 4 | 25 | 75 | 0 |
| 9 | 2400 | 112 | | 3 | | | | | | | | | | | | | | | | | 3 | 100 | 0 | 0 |
| 10 | 3060 | 63.6 | | 4 | | | | | | | | | 1 | | | | | | | | 5 | 80 | 20 | 0 |
| 11 | 1480 | 59.7 | | | | | | | | | | | | | | | | | | | 0 | 0 | 0 | 0 |
| 12 | 1660 | 54.3 | | | | | | | | | | | 2 | | | | | | | | 2 | 0 | 100 | 0 |
| 13 | 2800 | 63 | | 2 | | | | | | | 1 | | | | | | | | 1 | | 4 | 50 | 25 | 25 |
| 14 | 1900 | 57.6 | 1 | | 2 | 1 | | | | | | | | | | | | | | | 4 | 100 | 0 | 0 |
